# Knowledge, attitude, and practice status of ovarian reserve function among women of childbearing age: a latent profile analysis

**DOI:** 10.3389/fpubh.2025.1660116

**Published:** 2025-11-04

**Authors:** Yawen Wang, Fei Sun, Yue Qin, Jue Wang, Aiping Fu, Li Yuan, Xia Lei, Lin Zhou

**Affiliations:** ^1^School of Nursing, Zhejiang Chinese Medical University, Hangzhou, Zhejiang, China; ^2^Hangzhou First People’s Hospital, Hangzhou, Zhejiang, China

**Keywords:** ovarian reserve function, knowledge, attitude, practice, latent profile analysis

## Abstract

**Objectives:**

We aimed to explore the current status and latent profiles of knowledge, attitude, and practice (KAP) on ovarian reserve function among women of childbearing age and to identify the factors associated with these profiles.

**Methods:**

Using convenience sampling, women of reproductive age in the Hangzhou area of China were enrolled as study participants between March and May 2025. Data were collected using a demographic questionnaire and an ovarian reserve KAP instrument. A latent profile analysis was conducted on the ovarian reserve function KAP, and a multivariate logistic regression analysis was used to explore the influencing factors of different profiles.

**Results:**

A total of 333 women of reproductive age were included in the study. The ovarian reserve function KAP score was 55.00 ± 7.06 points, the knowledge dimension score was 9.23 ± 1.59 points, the attitude dimension score was 27.58 ± 3.74 points, and the practice dimension score was 18.19 ± 2.93 points. The data were divided into three latent profiles: high knowledge-high attitude-high practice type (39.04%), high knowledge-moderate attitude-moderate practice type (38.74%), and low knowledge-low attitude-low practice type (22.22%). Multivariate analysis identified age, occupation, and production history were factors influencing the different latent profiles of the ovarian reserve function KAP.

**Conclusion:**

The KAP on ovarian reserve function among women of childbearing age is at an intermediate level and exhibits significant variability across different groups. Relevant educators and healthcare professionals should develop personalized intervention plans tailored to the specific characteristics and influencing factors of each group to enhance overall KAP, thereby safeguarding reproductive health.

## 1 Introduction

Infertility is defined as the inability to achieve a clinical pregnancy after 12 months of regular unprotected intercourse (1). Approximately 17% of women of childbearing age worldwide are affected by infertility, which is more prevalent in developing countries (2). This condition elevates psychological and social stress in couples, creates financial strain for families, and even increases marital dissolution risk (3–5). Ovarian reserve serves as a key biomarker of female reproductive potential (6). Diminished ovarian reserve (DOR) refers to a reduction in the number or quality of oocytes, leading to the loss of normal reproductive potential in the ovaries and affecting female fertility, making it one of the primary causes of infertility (7).

Recently, socioeconomic development and shifting reproductive attitudes have increased the prevalence of delayed childbearing. Growing numbers of women prioritize career advancement and income stability over reproduction, electing to delay childbirth (8, 9). However, ovarian reserve function declines progressively with advancing female age. As women enter their mid-30s, the depletion of oocytes accelerates, accompanied by a decline in quality, which can easily lead to unintentional childlessness (10). Leridon’s model estimates that 14% of women will remain childless if they delay starting to try to conceive until the age of 35 and 34.8% if they delay until age 40 (11). Moreover, ovarian reserve function is closely related to environmental and lifestyle factors (12, 13). Therefore, early identification of modifiable ovarian reserve determinants, implementation of ovarian preservation strategies, and timely fertility planning are critical.

The knowledge, attitude, and practice (KAP) theory is a widely recognized behavioral intervention theory that can be used to explain health behaviors (14). It posits that women must first acquire knowledge of DOR and its modifiable determinants, and then form a positive attitude based on this understanding to promote behavioral change. Current research primarily focuses on ovarian reserve testing (anti-Mullerian hormone levels, antral follicle count, etc.) and fertility preservation (e.g., oocyte cryopreservation) (15–17), neglecting the gaps in women’s knowledge of ovarian reserve and their willingness to protect ovarian reserve function. Insufficient attention has been given to KAP status regarding ovarian reserve function in women of reproductive age. Although a prior cross-sectional study assessed ovarian reserve KAP among reproductive-age women in the Chongqing area of China (18), it directly judged levels based on scale scores, overlooking individual heterogeneity across demographic strata and failing to analyze population typologies underpinning KAP variations.

Latent Profile Analysis (LPA) is a human-centered method that categorizes research subjects into different latent categories by analyzing differences in their responses to overt items, thereby identifying heterogeneity within groups (19). This method aims to minimize individual differences within the same category while maximizing differences between categories, thereby improving classification accuracy and effectively capturing the typical characteristics of each subgroup (20). Therefore, this study employed LPA to explore the latent characteristics of ovarian reserve function in women of reproductive age and further analyzed the influencing factors of each subgroup, providing a reference basis for developing targeted intervention measures.

## 2 Methods

### 2.1 Study design and participants

This multicenter cross-sectional study employed convenience sampling to recruit 333 women of reproductive age from one hospital and two universities in Hangzhou, China, between 13 March and 29 May 2025. Inclusion criteria for this study were as follows: (1) age 18–48 years; (2) ability to correctly understand the questionnaire content and provide accurate responses; and (3) voluntary consent to participate in the survey. Exclusion criteria were as follows: (1) women working in the field of reproductive medicine and (2) those with severe medical conditions or cancer. According to the requirements of multiple regression analysis, the sample size should be at least 10–15 times the number of independent variables. This study included 15 independent variables. Considering a 20% rate of invalid questionnaires, the final calculated sample size required was at least 180 cases.

### 2.2 Measurement instruments

#### 2.2.1 Sociodemographic information

A self-designed questionnaire was used to collect sociodemographic data, including age, ethnicity, residence, monthly income per household, education, occupation, marital status, pregnancy history, production history, smoking, drinking, dietary habits, heavy makeup habits, family history of premature ovarian insufficiency (POI), and history of pelvic surgery.

#### 2.2.2 Ovarian reserve function KAP questionnaire

The ovarian reserve function KAP questionnaire, developed by Yuan et al. (18), was used to assess ovarian reserve function KAP among women of childbearing age. The knowledge dimension consisted of 11 questions, with 1 point awarded for correct answers and 0 points for incorrect answers or uncertainty. The attitude dimension consists of 7 questions, using a 5-point Likert scale from “strongly disagree” to “strongly agree,” scored 1–5 points respectively; question 6 is negative, scored 5–1 points, respectively, from “strongly disagree” to “strongly agree.” The practice dimension consists of 5 items, scored 1–5 points, respectively, from “very unwilling” to “very willing.” The standardized score for each dimension is calculated as the actual score divided by the total score multiplied by 100. A higher score indicates a higher level of KAP regarding the protection of ovarian reserve function in reproductive-age women. The original Cronbach’s *α* was 0.816; in our cohort, it was 0.840.

### 2.3 Statistical collection

All questionnaires were distributed via the Questionnaire Star online platform, using consistent instructions, including an introduction to the purpose and significance of the study, and instructions on how to complete the questionnaire. Participants were assured of privacy protection, and only after obtaining informed consent online could they proceed with completing the questionnaire. If participants had any questions or decided to withdraw midway, they could contact the researchers via WeChat. A total of 378 questionnaires were distributed in this study, with 333 valid responses collected, resulting in an effective response rate of 88.10%. This study was performed in accordance with the Declaration of Helsinki, and ethical approval was granted by the Ethics Committee of Hangzhou First People’s Hospital (Number: 2025KY070).

### 2.4 Statistical analysis

Statistical analysis was performed using SPSS 25.0 and Mplus 8.30. Firstly, categorical variables were presented as frequencies and percentages, while normal continuous variables were shown as Mean ± Standard deviation. Secondly, we constructed a latent profile model with standardized KAP dimension scores (knowledge, attitudes, practices) as continuous manifest variables. The process of LPA involves initiating with a one-class model and successively adding more classes, with parameters calculated for each model. The optimal model was selected based on fit indices, including the Akaike Information Criterion (AIC), Bayesian Information Criterion (BIC), adjusted Bayesian Information Criterion (aBIC), Entropy, the Lo–Mendell–Rubin test (LMRT), and the bootstrapped likelihood ratio test (BLRT). The smaller the AIC, BIC, and aBIC values, the better the model fit. The closer the Entropy is to 1, the more accurate the classification. Entropy > 0.8 indicates classification accuracy exceeding 90%. *p* < 0.05 values for LMRT and BLRT indicate that the k-category model is significantly superior to the k-1-category model. Thirdly, we performed a χ2 test or Fisher’s exact test to compare the characteristics of subgroups within the population and the Bonferroni method for multiple comparisons. The variables with statistical significance in univariate analysis were included in a multivariate analysis to identify the factors that influenced the latent profiles. A *p*-value of <0.05 indicated statistical significance.

## 3 Results

### 3.1 Characteristics of the participants

Among the 333 participants, 214 (64.26%) were under 25 years old, 78 (23.42%) were between 25 and 29 years old, and 41 (12.32%) were over 30 years old. More details are shown in Table 1.

**Table 1:** Characteristics of the participants (*N* = 333).

Characteristic	Number (%)
Age (years)	
18 ~ 24	214 (64.26)
25 ~ 29	78 (23.42)
≥30	41 (12.31)
Ethnicity	
Han	320 (96.10)
Others	13 (3.90)
Residence	
City	222 (66.67)
Non-city	111 (33.33)
Monthly income per household (yuan)	
<5000	112 (33.63)
5000 ~ 10000	120 (36.04)
10000 ~ 20000	73 (21.92)
>20000	28 (8.41)
Education	
Middle school/high school/technical secondary school and below	9 (2.70)
Junior college/university	261 (78.38)
Master’s degree and above	63 (18.92)
Occupation	
Regular employee	156 (46.85)
Others	177 (53.15)
Marital status	
Married	45 (13.51)
Others	288 (86.49)
Pregnancy history	
No	306 (91.89)
Yes	27 (8.11)
Production history	
No	311 (93.39)
Yes	22 (6.61)
Family history of POI	
Yes	4 (1.20)
No	233 (69.97)
Unclear	96 (28.83)
History of pelvic surgery	
Yes	18 (5.41)
No	315 (94.59)
Smoking	
Yes	2 (0.60)
No	331 (99.40)
Drinking	
Yes	23 (6.91)
No	310 (93.09)
Dietary habits	
Ordering food online	63 (18.92)
Restaurant/cafeteria	159 (47.75)
Cooking at home	111 (33.33)
Heavy makeup habits	
Yes	24 (7.21)
No	309 (92.79)

### 3.2 Ovarian reserve function KAP questionnaire

The total score for the Ovarian reserve function KAP questionnaire among 333 women of reproductive age was 55.00 ± 7.06 points. The knowledge dimension score was 9.23 ± 1.59 points, the attitude dimension score was 27.58 ± 3.74 points, and the practice dimension score was 18.19 ± 2.93 points. Among these three dimensions, the three items with the highest error rates and lowest scores are listed in Table 2.

**Table 2:** The top 3 items with the highest error rates/lowest scores in each dimension of ovarian reserve function KAP.

Item	Score rate (%) / score
Knowledge	
Immune diseases, such as systemic lupus erythematosus, may affect ovarian reserve function.	84.38%
The number of a person’s ova is determined at birth.	57.96%
Oral contraceptives do not decrease ovarian reserve function in women.	51.05%
Attitude	
I am willing to pay for ovarian reserve testing.	3.50 ± 0.88
I am concerned about the possible decrease in ovarian reserve function.	3.63 ± 0.92
I think that decreasing ovarian reserve function does not have an impact on my life, so I do not need to be concerned.	3.44 ± 1.06
Practice	
I am always in a good mood in my daily life.	3.69 ± 0.86
I avoid staying up late in my daily life.	2.89 ± 1.03
I eat vitamin-rich foods regularly.	3.47 ± 0.93

### 3.3 LAP of ovarian reserve function KAP

Using standardized KAP scores as indicators, latent profiles were fitted from one to five groups, labeled as Model 1 to Model 5 (Table 3). As the number of latent profiles increased, AIC, BIC, and aBIC all decreased gradually, and Entropy remained >0.8. To ensure the accuracy of model classification, each model must have at least 50 participants. Models 4 and 5 had insufficient sample sizes and were therefore unsuitable for classification. Therefore, this study selected Model 3 as the optimal fitting model. When the model had three profiles, AIC, BIC, and aBIC were relatively small, Entropy was > 0.8, and both LMRT and BLRT tests were significant at the *p* < 0.001 level. The posterior probability of the 3-profile model was 90.9 to 94.3%, indicating that this model was acceptable and had good discriminative power.

**Table 3:** Indicators of latent profile analysis for ovarian reserve function KAP.

Model	AIC	BIC	ABIC	Entropy	BLRT	VLMR	Category probability
1	7840.118	7862.967	7843.934	–	–	–	1.000
2	7556.268	7594.349	7562.628	0.940	<0.001	<0.001	0.769/0.231
3	7439.019	7492.333	7447.924	0.824	<0.001	<0.001	0.387/0.390/0.222
4	7385.079	7453.626	7396.529	0.865	0.017	<0.001	0.174/0.378/0.060/0387
5	7367.392	7451.171	7381.386	0.892	0.003	<0.001	0.009/0.174/0.051/0.375/0.390

**Figure 1:**
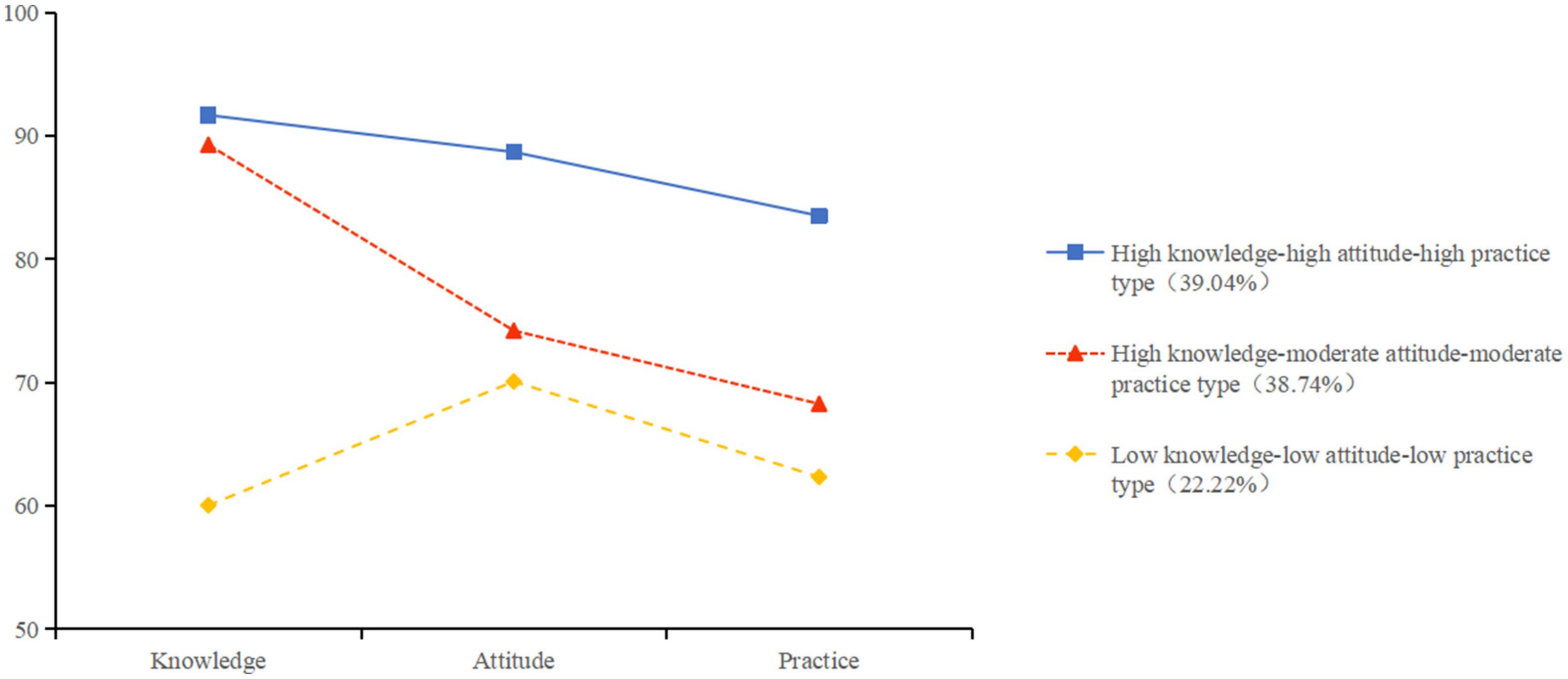
Distribution of characteristics among the three latent profiles of ovarian reserve function KAP. The vertical coordinate: standardized scores.

The three latent profiles were characterized and named according to their respective scores across the knowledge, attitude, and practice dimensions related to ovarian reserve function, as illustrated in Figure 1. Profile 1, termed the “high knowledge–high attitude–high practice type,” comprised 130 women who exhibited the highest scores across all three dimensions. Profile 2, labeled the “high knowledge–moderate attitude–moderate practice type,” included 129 women and was distinguished by relatively high knowledge scores, with attitude and practice scores falling between those of Profile 1 and Profile 3. Profile 3, identified as the “low knowledge–low attitude–low practice type,” consisted of 74 women who demonstrated the lowest scores in all dimensions.

### 3.4 Factors influencing ovarian reserve function KAP profiles in reproductive-age women

The results of the univariate analysis showed that age (*χ*^2^ = 48.120, *p* < 0.001), ethnicity (*χ*^2^ = 8.249, *p* = 0.016), residence (*χ*^2^ = 7.478, *p* = 0.024), monthly income per household (*χ*^2^ = 16.448, *p* = 0.012), education (Fisher = 24.367, *p* < 0.001), occupation (*χ*^2^ = 11.824, *p* = 0.003), marital status (*χ*^2^ = 21.310, *p* < 0.001), pregnancy history (*χ*^2^ = 29.030, *p* < 0.001), production history (*χ*^2^ = 35.009, *p* < 0.001), and history of pelvic surgery (*χ*^2^ = 16.730, *p* < 0.001) were significantly associated with KAP profiles. More details were shown in Table 4.

**Table 4:** Univariate analysis of latent profiles for ovarian reserve function KAP [*n* (%)].

Item	High knowledge-high attitude-high practice type (*n* = 130)	High knowledge-moderate attitude-moderate practice type (*n* = 129)	Low knowledge-low attitude-low practice type (*n* = 74)	*χ*^2^	*p*
Age (years)					
18 ~ 24	68 (52.31)	106 (82.17)	40 (50.05)	48.120	<0.001
25 ~ 29	46 (35.38)	19 (14.73)	13 (17.57)		
≥30	16 (12.30)	4 (3.10)	21 (28.38)		
Ethnicity					
Han	128 (98.46)	125 (96.90)	67 (90.54)	8.249	0.016
Others	2 (1.54)	4 (3.10)	7 (9.45)		
Residence					
City	97 (74.62)	82 (63.57)	42 (56.76)	7.478	0.024
Non-city	33 (25.38)	47 (36.43)	32 (43.24)		
Monthly income per household (yuan)					
<5000	35 (26.92)	43 (33.33)	34 (45.95)	16.448	0.012
5000 ~ 10000	42 (32.31)	54 (41.86)	24 (32.43)		
10000 ~ 20000	41 (31.54)	21 (16.28)	11 (14.86)		
>20000	12 (9.23)	11 (8.53)	5 (6.76)		
Education					
Middle school/high school/technical secondary school and below	0 (0.00)	0 (0.00)	9 (12.16)	24.367	<0.001
Junior college/university	101 (77.69)	107 (82.95)	53 (71.62)		
Master’s degree and above	29 (22.31)	22 (17.05)	12 (16.22)		
Occupation					
Regular employee	75 (57.69)	56 (43.41)	25 (33.78)	11.824	0.003
Others	55 (42.30)	73 (56.59)	49 (66.22)		
Marital status					
Married	23 (17.69)	4 (3.10)	18 (24.32)	21.310	<0.001
Others	107 (82.31)	125 (96.90)	56 (75.68)		
Pregnancy history					
No	123 (94.62)	126 (97.67)	57 (77.03)	29.030	<0.001
Yes	7 (5.38)	3 (2.33)	17 (22.97)		
Production history					
No	126 (96.92)	127 (98.45)	58 (78.38)	35.009	<0.001
Yes	4 (3.08)	2 (1.55)	16 (21.62)		
History of pelvic surgery					
Yes	3 (2.31)	4 (3.10)	11 (14.86)	16.730	<0.001
No	127 (97.69)	125 (96.90)	63 (85.14)		

Using the “high knowledge-high attitude-high practice type” as a reference, the statistically significant indicators from the univariate analysis were used as independent variables in the multivariable analysis. The results indicate that age, occupation, and production history are factors influencing ovarian reserve function KAP profiles in reproductive-age women (Table 5). Women aged 18–24 (vs. ≥30; 18–24, OR = 4.530, 95%CI: 1.016–20.194, *p* = 0.048) are more likely to belong to the “high knowledge-medium attitude-medium practice type.” Women aged 18–24 (vs. ≥30; 18–24, OR = 0.170, 95%CI: 0.042–0.688, *p* = 0.013), women aged 25–29 (vs. ≥30; 25–29, OR = 0.215, 95%CI: 0.057–0.814, *p* = 0.024), women in regular employment (OR = 0.195, 95%CI: 0.078–0.488, *p* < 0.001), and women without production history (OR = 0.045, 95%CI: 0.002–0.866, *p* = 0.040) are less likely to belong to “low knowledge-low attitude-low practice type.”

**Table 5:** Multivariate logistic regression analysis of influencing factors for latent profiles of ovarian reserve function KAP.

Item	*β*	SE	Wald *χ*^2^	*p*	OR	95%CI
High knowledge-moderate attitude-moderate practice type vs high knowledge-high attitude-high practice type^a^						
Age (years)^b^						
18 ~ 24	1.511	0.763	3.924	0.048	4.530	1.016 ~ 20.194
Low knowledge-low attitude-low practice type vs high knowledge-high attitude-high practice type^a^						
Age (years)^b^						
18 ~ 24	−1.769	0.712	6.174	0.013	0.170	0.042 ~ 0.688
25 ~ 29	−1.539	0.681	5.115	0.024	0.215	0.057 ~ 0.814
Occupation^c^	−1.633	0.467	12.232	<0.001	0.195	0.078 ~ 0.488
Production history^d^	−3.104	1.512	4.225	0.040	0.045	0.002 ~ 0.866

a High knowledge-high attitude-high practice type as the reference.

b Age ≥30 years old as the reference.

c Occupation is “others” as the reference.

d Delivery history is “yes” as the reference.

## 4 Discussion

This study showed that the total score of ovarian reserve function KAP was 55.24 ± 6.92 points, at a medium level. The knowledge and attitude dimensions were relatively high, exceeding the findings of Yuan et al. (18). This indicates that women have good understanding and positive attitudes toward ovarian reserve protection. Specifically, 83.2% of participants prioritized preventing reserve depletion, 85.6% valued regular monitoring, and 45.7% expressed testing cost willingness. O’Brien et al. (21) conducted a study on KAP regarding ovarian reserve testing among the general population of women of reproductive age and also found that 64.8% of the population held a positive attitude toward it, particularly younger women.

In contrast, practice competencies were deficient, particularly regarding lifestyle regulation. The item “I avoid staying up late in daily life” scored only 2.84 ± 1.07, with 36.63% of the participants frequently staying up late. Therefore, it is necessary to change their understanding of the close association between lifestyle and ovarian reserve function to improve their self-management levels. The item with the lowest success rate in the knowledge dimension was “Oral contraceptives do not cause a decrease of ovarian reserve function in women.” The use of hormonal contraceptives leads to a significant decrease in ovarian reserve parameters defined by anti-Mullerian hormone (AMH), antral follicle count (AFC), and ovarian volume (22, 23). Although some studies have found that this decline is reversible, with ovarian reserve markers returning to normal within 2 months of discontinuing the medication (24), women of reproductive age should be aware of the association between hormonal contraceptives and ovarian reserve function indicators. They should have their ovarian reserve function assessed before using hormonal contraceptives to identify early ovarian insufficiency (25). Healthcare professionals should prioritize personalized risk assessment and ovarian reserve screening for women using hormonal contraceptives to determine whether they have an increased risk of reduced reproductive lifespan.

Our study used latent profile analysis to divide reproductive-age women’s ovarian reserve function KAP into three latent profiles, indicating the presence of population heterogeneity. Among these, 22.22% of women were classified into the “low knowledge–low attitude–low practice type,” which was characterized by insufficient knowledge, negative attitudes, and inadequate protective practices. Women without regular employment were identified as being at significant risk of belonging to this profile. They may lack stable income and social security, facing significant economic and social pressures, which lead to insufficient attention to their reproductive health. Consistent with previous studies, women without regular employment demonstrated significantly lower knowledge scores regarding ovarian reserve function (18). Since knowledge forms the foundation for fostering positive attitudes and correct behaviors, insufficient knowledge may lead to more negative attitudes toward ovarian reserve protection and subsequently hinder the adoption of preventive practices. Therefore, policymakers and healthcare professionals should strengthen support for this group, including disseminating targeted ovarian health knowledge, improving access to reproductive health services, and promoting enhanced social security policies to alleviate their financial burdens.

Production history significantly influences the profile of ovarian reserve function KAP. Women with a history of childbirth exhibited a significantly elevated risk of belonging to the “low knowledge-low attitude-low practice” profile. This may occur because entering the post-reproductive phase leads them to deprioritize the maintenance of fertility and ovarian reserve, as they perceive their core reproductive goals as achieved (26). Importantly, ovarian reserve function decline not only compromises fertility but also associates with metabolic dysregulation, elevated cardiovascular risk, and premature perimenopause (27, 28), adversely impacting physical and psychological well-being. Consequently, establishing a comprehensive lifetime reproductive health management framework for parous women is imperative. Healthcare providers must integrate ovarian preservation into continuity care models, facilitating women’s transition from reproductive completion to lifelong health maintenance.

Interestingly, our study identified a distinct profile characterized by “high knowledge-moderate attitude- moderate practice.” This profile encompasses 38.74% of women, predominantly young women aged 18–24 years. The women in this age group are at the early stages of their academic or professional trajectories, during which educational and career advancement often takes priority (29). Although these women possess adequate knowledge of ovarian reserve function, their attitudes and practices remain underdeveloped. This discrepancy may stem from developmental needs and the perception that declining fertility rates represent a distant threat, leading to relatively weaker risk awareness of reproductive health (30). Previous studies have found that many women only initiate health behavior changes and reproductive choices after detecting abnormal clinical indicators of ovarian reserve function (31, 32). Furthermore, studies suggest that younger women may overestimate the efficacy of assisted reproductive technologies (ART) in overcoming future infertility, thereby underestimating the urgency of proactive ovarian health preservation (33). This mismatch between knowledge, attitude, and behavioral enactment illustrates a critical gap in health literacy. Therefore, clinical healthcare providers should prioritize bridging this gap by using feedback of clinical indicators to reinforce risk perception, correcting over-optimism regarding ART, and promoting the early adoption of protective behaviors among younger women.

The limitations of this study include its cross-sectional design, the restriction of participants to the Hangzhou area of China, and the distribution of questionnaires primarily in hospitals and universities. These factors may skew the results toward women with higher health literacy, thereby limiting the generalizability of the findings. Future studies should consider expanding the sample size to include women of reproductive age from diverse geographical and cultural backgrounds, conducting multi-center, large-scale studies to further validate the findings, and implementing longitudinal tracking to dynamically observe changes in women’s KAP regarding ovarian reserve function.

## 5 Conclusion

Women of reproductive age can be categorized into three latent profiles based on their ovarian reserve function KAP: high knowledge-high attitude-high practice type, high knowledge-moderate attitude-moderate practice type, and low knowledge-low attitude-low practice type. These profiles exhibit significant heterogeneity. Relevant educators and healthcare professionals should tailor interventions and support for women of reproductive age based on the characteristics and influencing factors of each profile to enhance their KAP regarding ovarian reserve function.

## Data Availability

The original contributions presented in the study are included in the article/supplementary material, further inquiries can be directed to the corresponding author.
